# Proteomic time course of breast cancer cells highlights enhanced sensitivity to Stat3 and Src inhibitors prior to endocrine resistance development

**DOI:** 10.1038/s41417-022-00548-0

**Published:** 2022-10-20

**Authors:** Stephen F. Madden, Mattia Cremona, Angela M. Farrelly, Weng Hei Low, Jean McBryan

**Affiliations:** 1 Data Science Centre, RCSI University of Medicine and Health Sciences, Dublin, D02 YN77 Ireland; 2 Dept Molecular Medicine, RCSI University of Medicine and Health Sciences, Beaumont Hospital, Dublin, D09 YD60 Ireland; 3 School of Medicine, Perdana University-RCSI, Serdang, Malaysia; 4 Dept Surgery, RCSI University of Medicine and Health Sciences, Beaumont Hospital, Dublin, D09 YD60 Ireland

**Keywords:** Breast cancer, Cell biology

## Abstract

To prevent the development of endocrine-resistant breast cancer, additional targeted therapies are increasingly being trialled in combination with endocrine therapy. The molecular mechanisms facilitating cancer cell survival during endocrine treatment remain unknown but could help direct selection of additional targeted therapies. We present a novel proteomic timecourse dataset, profiling potential drug targets in a population of MCF7 cells during 1 year of tamoxifen treatment. Reverse phase protein arrays profiled >70 proteins across 30 timepoints. A biphasic response to tamoxifen was evident, which coincided with changes in growth rate. Tamoxifen strongly impeded cell growth for the first 160 days, followed by gradual growth recovery and eventual resistance development. The growth-impeded phase was distinguished by the phosphorylation of Stat3 (y705) and Src (y527). Tumour tissue from patients treated with neo-adjuvant endocrine therapy (<4 months) also displayed increased Stat3 and Src signalling. Inhibitors of Stat3 (napabucasin) and Src (dasatinib), were effective at killing tamoxifen-treated MCF7 and T47D cells. Sensitivity to both drugs was significantly enhanced once tamoxifen had induced the growth-impeded phase. This novel proteomic resource identifies key mechanisms enabling cell survival during tamoxifen treatment. It provides valuable insight into potential drug combinations and timing that may prevent the development of endocrine resistance.

## Introduction

Endocrine resistance remains an unsolved clinical challenge in the treatment of breast cancer. Much has been learnt about the underlying molecular mechanisms, particularly with the help of cell lines that have acquired endocrine resistance. These discoveries have led to the development of many new targeted drugs for treating advanced, endocrine-resistant breast cancer patients. However, the subsequent development of multi-drug resistance, which frequently follows endocrine resistance, is obstructing our attempts to cure breast cancer. Enhancing our primary treatment of breast cancer to prevent rather than treat endocrine resistance is a necessity.

A number of drugs have been combined with endocrine therapy and are being tested in the adjuvant setting for the treatment of primary breast cancer to prevent disease recurrence. To date, the results of these trials have been mixed. For example, the CDK4/6 inhibitor, Abemaciclib, when combined with standard of care endocrine therapy for 2 years, demonstrated a significant improvement in invasive disease-free survival (DFS) compared to endocrine therapy alone (MonarchE trial) [1]. A similar trial with the CDK4/6 inhibitor ribociclib and endocrine therapy is currently underway with results awaited (NATALEE trial). However, trials with palbociclib, another CDK4/6 inhibitor, failed to demonstrate a significant benefit in the adjuvant setting (PENELOPE-B trial, PALLAS trial) [2, 3]. This is despite the fact that abemaciclib, palbociclib and ribociclib have all demonstrated significant progression-free survival benefit in the advanced breast cancer setting [4]. The mTOR inhibitor, everolimus, has also been tested in the adjuvant setting in combination with endocrine therapy (UNIRAD trial) [5]. No improvement in 3-year DFS was detected compared to endocrine therapy alone. This, despite the fact that everolimus has proven benefit in combination with endocrine therapy in the advanced breast cancer setting (BOLERO trials) [6]. Trial design, selection of high-risk patients for adjuvant trials and length of follow-up undoubtedly contribute to the mixed results seen so far. However, it is evident that the biology of primary tumour cells and their microenvironment is distinct to that of the metastatic tumour and not all drug treatments will achieve desired outcomes if transferred directly from one setting to the other. Identifying treatment combinations, which directly target the biology of primary tumour cells as they are treated with endocrine therapy, would be desirable.

Cell lines with acquired endocrine resistance have been heavily studied for their mechanisms of resistance, yet the process of acquiring resistance remains poorly understood. Such cell lines have been developed since the 1980s through long-term oestrogen withdrawal (e.g. LTED cells) [7–9], long-term tamoxifen treatment (e.g. TAMR-1 cells) [10–12], stable aromatase expression with long-term aromatase inhibitor treatment [13, 14], or a variety of other treatments and transfections which lead to endocrine resistance (eg. LCC9, LY2 cells) [15, 16]. The process of developing endocrine resistance can take anything from a few weeks to 18 months depending on treatment conditions such as drug concentration, stable or increasing drug dose, the presence of oestrogen or serum in the growth media and the methods used to determine resistance. Numerous groups have conducted pairwise comparisons of parental sensitive cells with acquired endocrine-resistant cells. However, omics analysis of the time-course of resistance development is a more novel approach to understanding the process of resistance development. Recently, two groups have looked at profiling the transcriptomics of acquiring endocrine resistance by conducting RNAseq or microarray analysis on MCF7 cells during the time-course of tamoxifen treatment and resistance development [17–19]. Both groups reported interesting gene expression dynamics and a critical transition from the pre-resistant state to the drug-tolerant stage. The need to intervene with treatment prior to this tipping point was reported although both groups focussed their studies on prognostic biomarkers rather than treatment options. To the best of our knowledge, a detailed proteomic time-course of endocrine resistance development has not previously been reported.

In this study, we sequentially monitor MCF7 and T47D cells as they are exposed to tamoxifen and acquire resistance. We conduct proteomic profiling of the time course of tamoxifen treatment, identifying a biphasic response in the development of resistance. We highlight targetable proteins and phospho-proteins that are specifically regulated during the dormant-like period of impeded growth, before the transition to endocrine resistance. We provide supporting evidence that these protein pathways are also regulated in primary breast cancer patients in response to both tamoxifen and aromatase inhibitors. Finally, we demonstrate that drugs targeting these proteins have enhanced potency if delivered during the growth-impeded phase, encouraging the use of these targeted therapies in conjunction with endocrine therapy in the neoadjuvant and adjuvant setting for primary breast cancer.

## Materials and methods

### Cell lines and tamoxifen treatment

MCF7 cells (ECACC) were cultured in Eagle’s Minimum Essential Media supplemented with 10% FBS, L-glutamine and PenStrep. T47D cells (ECACC) were cultured in RPMI-1640 supplemented with 10% FBS, PenStrep and 0.1% human insulin. All cells were incubated at 37 °C with 5% CO_2_. Cell lines were authenticated by STR profiling and routinely tested for mycoplasma contamination.

To generate tamoxifen resistance, 10^−6 ^M 4-hydroxy-tamoxifen (4-OHT) was added to the culture media. Images were captured at regular intervals with a Nikon Eclipse TS100 microscope to monitor changes in cell appearance. Growth assays and hormone response assays (Supplementary Fig. S1) were conducted at regular intervals to monitor changes in growth and drug sensitivity. For omics analysis, one batch of MCF7 cells was serially cultured without interval until tamoxifen resistance developed. Cell pellets were collected at regular intervals for protein isolation. For western blot and toxicity assays, cells were paused at different stages of the tamoxifen response and frozen to liquid nitrogen. Cells were subsequently thawed and grown again, enabling cells from multiple phases to be grown at the same time and compared within the one assay. For toxicity assays, two additional batches of MCF7 cells were serially cultured with tamoxifen to achieve the growth-impeded phase.

### Growth assays

Twenty thousand cells/well were seeded in triplicate into 12-well plates in their standard culture media. Media was replaced every 2–3 days. On day 7, cells were trypsinised and live cells were manually counted using Trypan Blue. Cell counts were compared between assays to assess changes in growth rates.

### Real-time PCR

RNA was isolated from cell pellets using Qiagen RNeasy Mini kits with DNase treatment. cDNA was synthesised with Promega Im-Prom-II reverse transcriptase kit. Real-time PCR was conducted on the 7500HT-Fast Applied Biosystems machine using TaqMan PCR master mix (Biosciences, #4440047) and specific TaqMan probes (ESR1, Hs01046816_m1; ACTB, Hs01060665_g1) or Fast SYBR green PCR master mix with specific primers for PGR (PGR Fwd: AAATCATTGCCAGGTTTTCG, PGR Rev: TGCCACATGGTAAGGCATAA). The ΔΔCt method was used to calculate relative gene expression, normalised to β-actin.

### Protein isolation and western blot analysis

Protein was isolated from cell pellets using RIPA buffer supplemented with protease and phosphatase inhibitors (Roche, #11836170001, #4906845001). Protein extraction lysates were quantified and normalised using a bicinchoninic acid assay (DC Protein Assay, Bio-Rad). 30–40 μg total protein samples were size-separated by SDS PAGE using an 8% polyacrylamide gel. Following semi-dry transfer, nitrocellulose membranes were probed using antibodies detailed in Supplementary Table S1 or β-actin (A5316, Sigma). Chemiluminescence was visualised using the Amersham Imager 600 and quantified using Image J software. Six biological replicates from each phase were analysed. Protein from an untreated cell line, the growth-impeded phase, and the growth-recovery phase were grouped into one replicate set and loaded on the same gel to enable comparison. Densitometry values were normalised to β-actin and to the untreated cell line of each biological replicate set to aid visualisation on the graph. Raw densitometry values were statistically analysed using the Wilcoxon signed rank test.

### Reverse phase protein array (RPPA)

Briefly, a 2470 Arrayer (Aushon BioSystem, MA, USA) created a sample array on Oncyte Avid nitrocellulose-coated slides (Grace Bio-Labs, OR, USA). Immunostaining was performed on an automated slide stainer (Dako Link 48 – Agilent Technologies, CA, USA). Full details are provided in Supplementary Methods and raw data is provided in Supplementary Table S2.

### Toxicity assays

Two thousand cells/well were seeded into 96 well plates with technical triplicates for each condition. The following day, media was replaced with media containing 10^−6 ^M 4-OHT and napabucasin (0–3 µM) or dasatinib (0–20 µM). An appropriate amount of vehicle (DMSO) was added to ensure that all cells within an assay were treated with the same concentration of DMSO. Cells from the untreated cell line, the Growth-Impeded Phase and the Growth Recovery Phase were assayed at the same time so that the same drug preparation could be given to all 3 cell phases. Triplicate wells were treated with each drug concentration (technical replicates). After 5 days, CellTitre 96 Aqueous One Solution (Promega) was added as per the manufacturer’s instructions. Following 3 h incubation, absorbance was read at 490 nm on a Perkin Elmer, VictorX3 2030 Multilabel reader. Using the average of triplicate well absorbance readings, the percentage survival was calculated for each drug dose in each cell type. The mean absorbance from wells with no drug was regarded as 100% survival and the mean absorbance from blank wells (containing reagents but no cells) was regarded as 0% survival. The entire assay was repeated with 7–8 biological replicates and an IC50 value was calculated for each biological replicate using CompuSyn software [20]. With this information plots with error bars were generated and the data used is available in Supplementary Table S3 (the results from averaging the technical triplicates). Comparisons of IC50 values between groups (untreated, growth-impeded, growth recovery) were performed using a Welch two-sample *t*-test.

### Bioinformatic analysis and statistics

The RPPA data was visualised using principal component analysis (PCA). Based on the plot, the data was placed into three overarching groups: untreated (*n* = 5 replicate cell samples), growth-impeded (*n* = 15) and growth recovery (*n* = 14). Each of the 72 proteins/protein variants were compared using a *t*-test across consecutive groups (untreated vs growth-impeded, growth-impeded vs growth recovery) and *p*-values were adjusted using the Benjamini-Hochberg method [21]. No fold-change cut-off was applied.

Normalised gene expression data was downloaded from the gene expression omnibus (https://www.ncbi.nlm.nih.gov/geo/, accession numbers, GSE20181 and GSE147271). Differentially regulated gene lists were generated using the limma [22] package in R, based on the comparison of pre versus post treatment with either a neoadjuvant aromatase inhibitor or tamoxifen. A fold change of 2 and an adjusted *p*-value of 0.05 was considered significant. Enrichment for Stat3 target genes (*n* = 64) and dasatinib index genes (*n* = 124) was calculated using a fisher exact test in the differential gene expression lists for each gene expression dataset. All statistical calculations were carried out in the R statistical environment (https://www.r-project.org/).

## Results

### Proteomic analysis of the development of tamoxifen resistance in MCF7 cells: a biphasic response

**Fig. 1:**
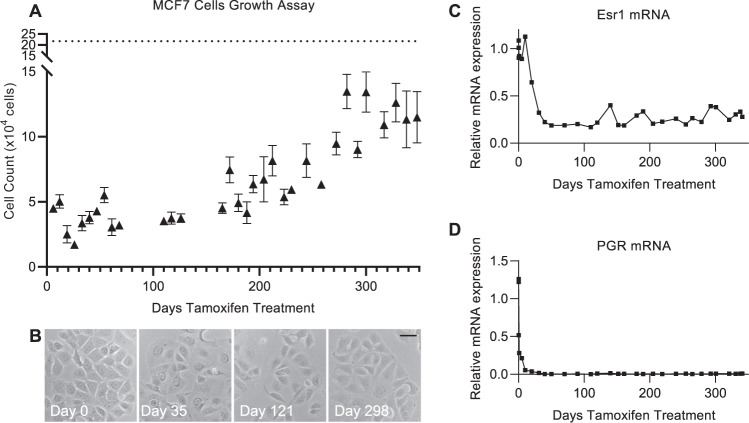
MCF7 cells acquire tamoxifen resistance without reactivating ERalpha. **A** Graph shows the results of 32 growth assays in MCF7 cells exposed to 10^−6 ^M 4-OHT for varying times (0–350 days, *x*-axis) prior to initiation of the growth assay. On each occasion, 20,000 cells were seeded in triplicate wells and manual cell counts were conducted 7 days later (*y*-axis). Data points represent mean +/− SEM with *n* = 3 for each individual assay. Dotted line represents the mean cell count for untreated MCF7 cells (*n* = 10 assays). **B** Micrographs of MCF7 cells following varying number of days of tamoxifen treatment, scale bar is 0.2 µm. **C** Real-time PCR analysis showing expression of oestrogen receptor (ESR1) over the course of 1 year of tamoxifen treatment in MCF7 cells. Expression is normalised to β-actin. **D** Real-time PCR analysis showing expression of progesterone receptor (PGR) over the course of 1 year of tamoxifen treatment in MCF7 cells. Expression is normalised to β-actin.

To generate tamoxifen resistant breast cancer cells, MCF7 cells were treated with a constant dose of 10^−6 ^M 4-hydroxy tamoxifen. The timing of resistance development was monitored at frequent intervals with growth assays (Fig. 1A), microscopically (Fig. 1B) and with hormone response assays (Supplementary Fig. S1). The growth rate of MCF7 cells was suppressed within the first 7 days of tamoxifen treatment, dropping to less than a quarter of the original growth rate. This slow growth rate was maintained for ~160 days before gradually starting to recover. The cells were deemed tamoxifen resistant when the growth rate levelled off and stabilised. This occurred almost 1 year after initiation of tamoxifen treatment and the resistant cells had a growth rate ~70% that of the parental MCF7 cells (Fig. 1A).

Ligand-independent oestrogen receptor activation, often arising through ESR1 gene mutation, is a well-documented mechanism of endocrine resistance, even though it exists in just a small minority of endocrine-resistant patients. Real-time PCR analysis of our cell line throughout the first year of tamoxifen treatment revealed a sustained decline in both ESR1 gene expression and the expression of the oestrogen-responsive gene PGR (Fig. 1C, D). Thus, representative of the majority of endocrine-resistant patients, an altered mechanism of oestrogen receptor activation does not appear responsible for the development of tamoxifen resistance in this model system.

**Fig. 2:**
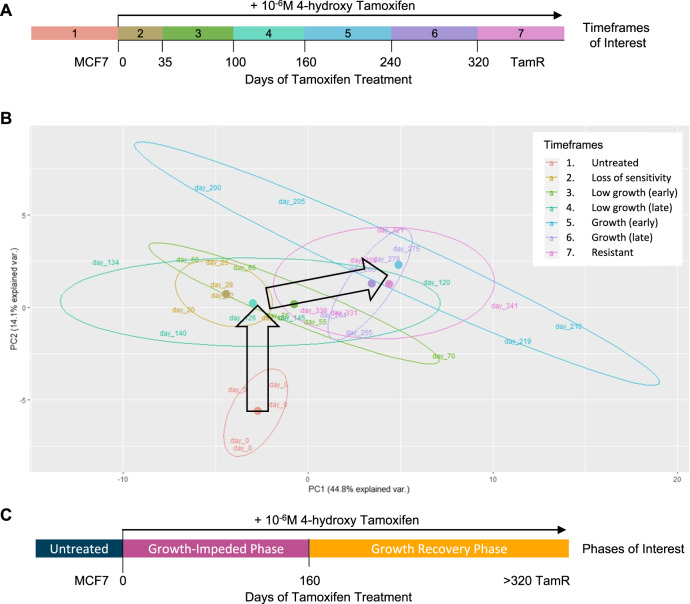
Biphasic proteomic response to long-term tamoxifen treatment. **A** Diagram representing 7 timeframes of interest that were selected before proteomic analysis of MCF7 cells treated long-term (up to 1 year) with tamoxifen. Timeframes were selected based on changes in growth rate and defined by the number of days of tamoxifen treatment as follows: 1, untreated MCF7 cells; 2, loss of sensitivity 0–35 days; 3, low growth (early) 35–100 days; 4, low growth (late) 101–160 days; 5, growth (early) 161–240 days; 6, growth (late) 241–320 days; 7, resistant >320 days. **B** PCA plot of the proteomic data with each data point labelled by the number of days with which MCF7 cells had been treated with tamoxifen. Coloured ovals group samples from each timeframe with coloured circles representing the central point of each timeframe. The first timeframe (untreated MCF7 cells) was the only one to cluster in isolation (at the base of the vertical arrow). Timeframes 2, 3 and 4 were heavily overlapping (centred close to the tip of the vertical arrow). Timeframes 5, 6 and 7 also overlapped (centred near the tip of the horizontal arrow). Large black arrows summarise the key trends observed in the proteomic profiles. **C** Diagram representing the reclassification of MCF7 tamoxifen response into phases of interest based on growth assays and proteomic data. Untreated cells enter a growth-impeded phase upon treatment with tamoxifen and subsequently enter a growth recovery phase before resistance develops.

To explore changes in protein expression during the development of tamoxifen resistance, reverse phase protein arrays (RPPA) were used to study cells at different stages of tamoxifen treatment. The arrays assessed the expression of 72 cancer-related proteins and phospho-proteins (full details in Supplementary Table S1). To facilitate in depth analysis, the development of tamoxifen resistance was divided into seven timeframes of interest (Fig. 2A) based on the results of the growth analysis. Five biological replicates from each of the 7 timeframes were arrayed. Data obtained from 34 of the 35 cell samples passed quality control tests with one sample (Day 210, Timeframe 5) being lost to protein degradation during sample preparation.

Principal component analysis (PCA) of the proteomic array data provided valuable insight to the dynamic process of tamoxifen resistance development (Fig. 2B). Timeframe 1 (untreated MCF7 cells) was the only timeframe to form a distinct cluster, separate to all other samples. Despite overlap between many of the tamoxifen-treated samples, those from Timeframes 2, 3 and 4 centred in a distinct location on the PCA plot compared to samples from Timeframes 5, 6 and 7 (Fig. 2B). The proteomic data thus revealed two major transitions in protein expression during tamoxifen treatment (represented by large black arrows in Fig. 2B). The first transition was triggered by the initiation of tamoxifen treatment and corresponded with the cells entering a growth-impeded phase in response to tamoxifen. The second transition in protein expression occurred between Timeframes 4 and 5 (after ~160 days of tamoxifen treatment) and coincided with the cells regaining the ability to grow in the presence of tamoxifen. The trigger for this second transition is unclear and there were no obvious changes in external cell culturing methods at this time. With this additional proteomic knowledge, the process of resistance development was reclassified for subsequent experiments into untreated phase, growth-impeded phase, and growth recovery phase, as detailed in Fig. 2C.

### Dynamic proteomic responses to tamoxifen

Having observed a biphasic response to long-term tamoxifen treatment, we were particularly interested to understand what keeps the cells alive during the growth-impeded phase. Historically, this phase has been overlooked as pairwise comparisons have most commonly been made between sensitive and resistant cells only. Our proteomic data suggest that the proteins involved in maintaining cell survival during the growth-impeded phase may be distinct to those involved in maintaining cell survival prior to tamoxifen treatment or following the development of tamoxifen resistance. The proteins and phospho-proteins significantly differentially regulated as cells enter the growth-impeded phase and re-emerge to growth recovery are listed in Tables 1 and 2 respectively. Complete, raw data from the RPPA analysis is available in Supplementary Table S2.

**Table 1:** Differentially expressed proteins as cells enter growth-impeded phase.

Protein	Fold change ctrl vs slow	Adjusted *p*-value
*Upregulated as cells enter growth-impeded phase*		
GAB1	1.63	<0.001
**STAT3**	1.37	0.033
MTOR (S2448)	1.31	0.035
p38 MAPK	1.28	0.001
CYCS	1.27	0.005
FAK	1.25	0.005
**STAT3 (Y705)**	**1.21**	**0.035**
BCLXL	1.18	0.035
PDK1	1.15	0.022
BCL2 (T56)	1.14	0.035
*Downregulated as cells enter growth-impeded phase*		
IGF1R	−2.02	<0.001
p27	−1.56	0.006
GSK3B (S9)	−1.45	<0.001
SHC (Y317)	−1.42	0.007
AKT (S473)	−1.36	0.035
PI3Kp110alpha	−1.35	0.004
PARP1	−1.21	0.005

Proteins with significant expression change in MCF7 cells treated with tamoxifen for 18–145 days (Timeframes 2, 3, 4) compared to untreated MCF7 cells (Timeframe 1). Proteins highlighted in bold were selected for further analysis.

**Table 2:** Differentially expressed proteins as cells enter growth recovery phase.

Protein	Fold change slow vs fast	Adjusted *p*-value
*Upregulated as cells enter growth recovery phase*		
GSK3B	1.73	0.012
BID	1.69	0.012
HIF1A	1.69	0.028
RSK	1.68	0.033
AKT	1.67	0.022
RSK (s380)	1.66	0.031
p53	1.64	0.040
SMAC/DIABLO	1.64	0.006
PKCalpha	1.64	0.014
PHD1	1.63	0.039
VEGFR2	1.54	0.033
MTOR (S2481)	1.53	0.041
**SRC**	**1.45**	**0.033**
CA9	1.44	0.033
p27	1.38	0.007
*Downregulated as cells enter growth recovery phase*		
p70 S6 Kinase (T389)	−1.95	0.002
AMPK (T172)	−1.64	<0.001
HER3 (Y1289)	−1.54	0.028
**STAT3 (Y705)**	**−1.54**	**0.001**
MET (Y1234/1235)	−1.48	0.033
CASP7 cleaved (D198)	−1.42	0.002
BCL2	−1.37	0.007
AKT (T308)	−1.34	0.001
AKT (S473)	−1.34	0.001
CASP3	−1.33	0.010
MCL1	−1.33	0.007
GSK3B (S9)	−1.32	0.009
CHEK1	−1.31	0.011
BCL2 (S70)	−1.29	0.005
RAF1 (S338)	−1.28	0.014
**SRC (Y527)**	**−1.27**	**0.003**
XIAP	−1.26	0.024
**SRC family (Y416)**	**−1.26**	**0.033**
FAK (Y925)	−1.23	0.002
CASP9 cleaved (D330)	−1.19	0.033
RAF1	−1.11	0.041

Proteins with significant expression change in MCF7 cells treated with tamoxifen for >160 days (Timeframes 5, 6, 7) compared to those treated for 18–145 days (Timeframes 2, 3, 4). Proteins highlighted in bold were selected for further analysis.

A total of 17 proteins were significantly differentially regulated as cells entered the growth-impeded phase (Table 1) while 36 significant expression changes were detected as cells entered growth recovery (Table 2). Members of the PI3K/AKT signalling pathway (PI3Kp110alpha, AKT S473) and phosphorylated GSK3B (S9) were significantly downregulated as cells entered the growth-impeded phase (−1.35, −1.36 and −1.45 fold changes respectively) and maintained relatively low expression in the resistant cells. By contrast, mTOR phosphorylation (S2448) displayed a significant and sustained increase in response to tamoxifen (+1.31 fold change). Significant changes in apoptotic regulators were also detected such as increased BCLXL (+1.18 fold change) and BCL2 (T56) (+1.14 fold change) during the growth-impeded phase. Though the biological relevance of these small fold changes would need to be confirmed, they may indicate potential protection against apoptosis at this time.

### Enhanced phosphorylation of Stat3 and Src in response to tamoxifen

**Fig. 3:**
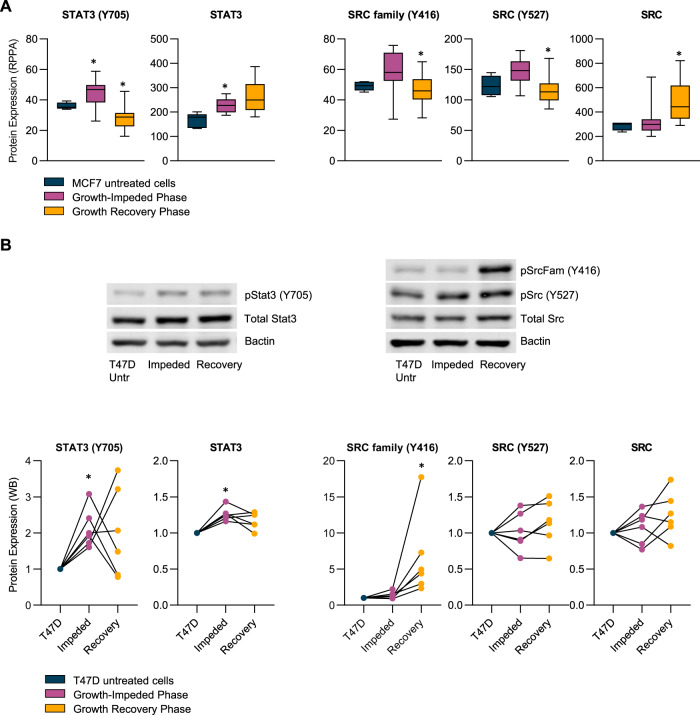
Dynamic regulation of Stat3 and Src proteins during the timecourse of tamoxifen treatment in MCF7 and T47D cells. **A** Min/max boxplots represent the relative protein expression as measured by RPPA analysis in MCF7 cells treated with tamoxifen for up to 1 year. Data from 5 antibodies are presented: STAT3 (Y705), STAT3, SRC family (Y416), SRC (Y527) and SRC. Navy: untreated MCF7 cells (*n* = 5), pink: cells at timepoints during the growth-impeded phase (*n* = 15), yellow: cells at time points during the growth recovery phase (*n* = 14). * Indicates significant expression regulation compared to the previous growth phase (*p* < 0.05). **B** Representative western blot images and densitometry analysis (*n* = 6 biological replicates) of T47D cells untreated (navy) and following tamoxifen treatment to induce a Growth-Impeded Phase (35–100 days, pink) or a Growth Recovery Phase (>100 days, yellow). Data for the same five antibodies for total and phosphorylated Stat3 and Src are presented, normalised to β-actin and to the untreated T47D controls. Each replicate set of samples run on the same gel are connected by a line in the scatter plots. * Indicates significant expression regulation compared to the previous phase (*p* < 0.05).

Two proteins, which make multiple appearances in Tables 1 and 2 particularly, attracted our attention. Stat3 is a transcription factor known for its involvement in stem cell self-renewal. Both Stat3 (+1.37 fold change, adjusted *p*-value = 0.033) and phospho Stat3 (Y705) (+1.21 fold change, adjusted *p*-value = 0.035) were significantly upregulated as cells entered the growth-impeded phase. Phospho Stat3 was subsequently downregulated as cells entered growth recovery (−1.54 fold change, adjusted *p*-value = 0.001) (Fig. 3A). This dynamic regulation suggested to us that Stat3 may play a distinct functional role during the slow-growing survival phase. The tyrosine kinase Src displayed a similar pattern of dynamic expression. An increase in phospho Src at both Y416 and Y527 sites during the growth-impeded phase (although not significant by RPPA) was followed by a significant decrease in phospho Src as cells entered growth recovery (Y416 site: −1.26 fold change, adjusted *p*-value = 0.033; Y527 site: −1.27 fold change, adjusted *p*-value = 0.003). Total Src levels significantly increased as cells recovered their growth rate (+1.45 fold change, adjusted *p*-value = 0.033). The dynamic regulation of both Src and Stat3 in MCF7 cells is shown in Fig. 3A.

We next established tamoxifen resistance in a separate, independent cell line, T47D cells. These cells achieved resistance quicker than the MCF7 cells but again a biphasic growth response was observed: first an extended period when tamoxifen was impeding growth (lasting ~100 days), followed by growth recovery to achieve a resistant state by ~250 days of tamoxifen treatment (Supplementary Fig. S2). Using Western blot analysis, we explored the protein expression and phosphorylation status of Stat3 and Src in T47D cells before tamoxifen treatment, during the growth-impeded phase and during the growth recovery phase (Fig. 3B). As with MCF7 cells, a significant increase was observed during the Growth-Impeded Phase of tamoxifen response in T47D cells for both phospho Stat3 (Y705) (+2.12 fold change, *p*-value = 0.031) and total Stat3 (+1.26 fold change, *p*-value = 0.031) (Fig. 3B). The expression and phosphorylation pattern of Stat3 was more variable moving from the growth-impeded phase to the growth recovery phase in the T47D model than it was in the MCF7 model with no significant pattern of change detected by Western blot. The pattern of Src phosphorylation was different between T47D and MCF7 models but significant regulation of Src phosphorylation was detected in both cell lines in response to tamoxifen (Fig. 3). In the T47D model, phospho Src (Y416) displayed increased expression in the Growth Recovery Phase compared to the previous phase. (+4.56 fold change, *p*-value = 0.031). Phospho Src (Y527) and total Src displayed some fluctuations in expression in the T47D model but no pattern of significant change.

### Enhanced sensitivity to napabucasin and dasatinib during the growth-impeded phase of tamoxifen response

**Fig. 4:**
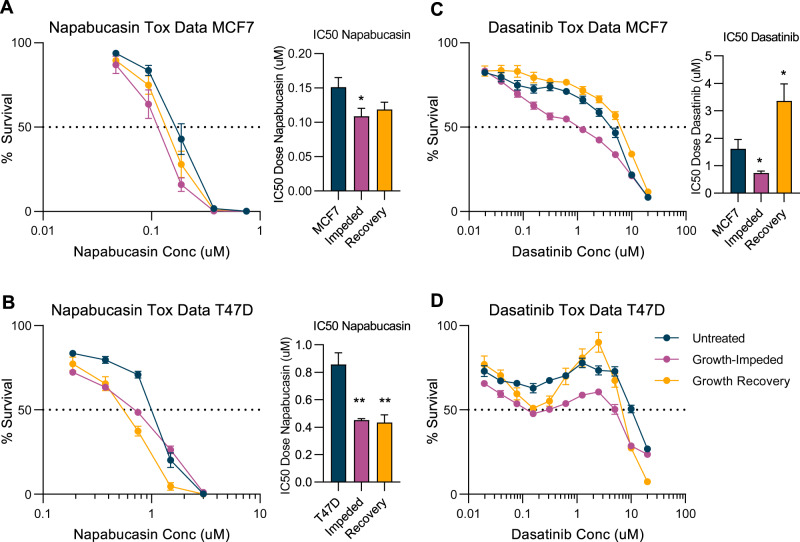
Enhanced sensitivity to Stat3 and Src inhibitors during the growth-impeded phase of tamoxifen response. Log dose response curves of targeted inhibitors in untreated parental cells (navy lines), cells pre-treated with tamoxifen to induce the growth-impeded phase (pink lines) and cells pre-treated with tamoxifen to induce the growth recovery phase (yellow lines). All cells were treated with 10^−6 ^M 4-OHT and variable doses of targeted drug during the 5 day toxicity assay. Curves show the mean percentage survival +/− SEM from 7 to 8 biological repeats. Bar charts show the IC50 values for cells at each phase (mean +/− SEM). **A** Parental cells are MCF7, targeted drug is napabucasin, a Stat3 inhibitor, *n* = 8 biological replicates from each phase. **B** Parental cells are T47D, targeted drug is napabucasin, *n* = 7 biological replicates from each phase. **C** Parental cells are MCF7, targeted drug is dasatinib, a Src inhibitor, *n* = 7 biological replicates from each phase. **D** Parental cells are T47D, targeted drug is dasatinib, *n* = 7 biological replicates from each phase. *significantly different to parental cells with *p* < 0.05 **significantly different to parental cells with *p* < 0.01.

The dynamic expression and activation of Stat3 and Src during the response to tamoxifen suggests that inhibiting these proteins might prevent breast cancer cell survival and the development of tamoxifen resistance. A number of Stat3 inhibitors have been developed and are being tested in clinical trials including the small molecule inhibitors napabucasin (multiple phase III trials), TTI-101 (phase I trial) and the antisense oligonucleotide AZD9150 (phase Ib/II trials) [23]. We conducted toxicity assays in MCF7 and T47D cells treated with tamoxifen and found that napabucasin was very effective at killing both cell lines (Fig. 4 and Supplementary Table S3). In MCF7 cells, the IC50 was calculated as 0.151 µM ± 0.014. Pre-treatment with tamoxifen to induce the growth-impeded phase significantly reduced the IC50 value to 0.109 µM ± 0.012 (*p*-value = 0.034). Cells in the growth recovery phase also had enhanced sensitivity to napabucasin compared to parental MCF7 cells with an IC50 of 0.119 µM ± 0.011 (*p*-value = 0.084) (Fig. 4A). Although phospho Stat3 (Y705) expression was reduced in the Growth Recovery Phase, total Stat3 levels remained high and may explain the maintained sensitivity. The reduction of IC50 values is not purely due to synergy with tamoxifen as all cell lines, including the parental MCF7 cells, were treated with both tamoxifen and napabucasin during this assay. Rather, the pre-treatment with tamoxifen is priming the cells to be sensitive to napabucasin, as anticipated from analysis of our RPPA data.

This toxicity assay was repeated in the independent T47D cell model with comparable results (Fig. 4B). T47D cells are less sensitive to napabucasin than MCF7 cells with an IC50 value of 0.857 µM ± 0.085. This is nonetheless still within the range of plasma concentrations achieved in patients during Phase 1 clinical trials of napabucasin [24]. Pre-treatment with tamoxifen almost halved the dose required to kill 50% of cells with the IC50 value significantly reducing to 0.451 µM ± 0.011 (*p*-value = 0.003) in growth-impeded phase T47D cells and to 0.434 µM ± 0.056 (*p*-value = 0.002) in T47D cells during the growth recovery phase.

Research into the inhibition of Src and other tyrosine kinases has been heavily driven by the leukemia research field and has led to the licensing of a number of Src inhibitors including dasatinib, Saracatinib, Bosutinib and Ponatinib. We selected dasatinib, one of the most widely studied Src inhibitors for our Toxicity assays. Dasatinib has entered clinical trials in breast cancer and has proven safety records if given in combination with endocrine therapy [25]. MCF7 cells have previously been classed as dasatinib-resistant by comparison to other cancer cell lines [26] and our toxicity assays measured an IC50 value of 1.617 µM ± 0.343. However, when we pre-treated MCF7 cells with tamoxifen to induce the growth-impeded phase, the cells became significantly more sensitive to dasatinib, achieving an IC50 value of 0.731 µM ± 0.069 (*p*-value = 0.042). Consistent with our RPPA data, the enhanced Src inhibitor sensitivity was confined to the growth-impeded phase as cells in the growth recovery phase lost dasatinib sensitivity, presenting an IC50 value of 3.359 µM ± 0.619 (*p*-value = 0.035) (Fig. 4C). In contrast to napabucasin, which produced 0% survival rates within the range of our assay, dasatinib was unable to kill all cells even at doses 10-fold the IC50 values.

Dasatinib is a multi-kinase inhibitor with high sensitivity for Src (enzyme IC50 0.5 nM) but it also targets BCR-ABL (IC50 < 1 nM), c-KIT (IC50 5 nM) and other tyrosine kinases [27]. This was highlighted when we conducted toxicity assays with dasatinib in T47D cells (Fig. 4D). Once again, pre-treatment with tamoxifen appeared to enhance sensitivity to dasatinib in these cells. However, at higher doses, dasatinib caused less cell death in these cells than at lower doses. The calculation of IC50 values was therefore unreliable as the 50% survival line was crossed at more than one dose. One possible explanation for these unusual looking dose response curves, is that inhibition of Src at relatively low doses, leads to cell death but inhibition of other tyrosine kinases by dasatinib at higher doses may actually provide a survival benefit to these cells.

### Regulation of Stat3 and Src-associated genes in primary breast cancer patients following neoadjuvant endocrine treatment

**Fig. 5:**
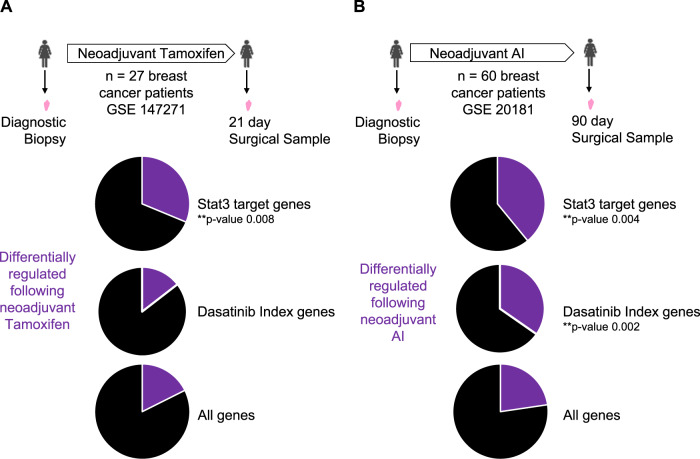
Differential expression of Stat3 and Src target genes in patients treated neoadjuvantly with either tamoxifen or aromatase inhibitor. **A** Schematic overview of a transcriptomic data set (GSE147271) containing matched samples from 27 primary breast cancer patients before and after 21 days of neoadjuvant tamoxifen treatment. Pie charts show the proportion of genes which are differentially regulated following neoadjuvant treatment (purple segment). Stat3 target genes are a pre-defined set of 64 genes relevant to human cancers. Dasatinib Index genes are a pre-defined set of genes derived from breast cancer cell lines. **B** Schematic overview of a transcriptomic data set (GSE20181) containing matched samples from 60 breast cancer patients before and after 90 days of neoadjuvant aromatase inhibitor treatment. Pie charts show the proportion of genes differentially regulated following neoadjuvant treatment as described in part **A**.

Exploring how patient tumours respond over time to tamoxifen therapy is notoriously challenging in the clinic as traditionally tumours are surgically removed before endocrine therapy is commenced. One opportunity arises when neoadjuvant therapy is delivered and tumours are sampled both before and after therapy. We assessed one such dataset (GSE147271) where 27 breast cancer patients received tamoxifen for 21 days prior to surgery [28]. Gene expression analysis was conducted on pre-treatment diagnostic biopsies and on post-treatment surgical samples. Approximately 1/6 of all genes assayed were differentially expressed following tamoxifen treatment (2875 of 16,320 total genes). As a measure of Stat3 activity, we assessed the expression of a published panel of 64 pre-defined Stat3 target genes which have been confirmed by either Electrophoretic Shift Mobility Assay or Chromatin Immunoprecipitation [29]. We identified an enrichment of differentially expressed genes amongst the Stat3 targets: ~1/3 of Stat3 target genes were differentially regulated following neoadjuvant tamoxifen (20 of 64 Stat3 target genes), which is significantly more than would be expected by chance (*p*-value = 0.008) (Fig. 5A). The direction of gene expression change (upregulation or downregulation) is often highly dependent on the setting and timing. Nonetheless 70% of differentially regulated Stat3 target genes changed in response to neoadjuvant tamoxifen treatment in a direction consistent with previous reports of Stat3 activation in other settings [29, 30]. A second dataset (GSE20181) was also examined, this time where 60 breast cancer patients were treated with neoadjuvant aromatase inhibitor (AI) for 90 days prior to surgery [31, 32]. Once again, a significant enrichment in the number of Stat3 target genes that were differentially regulated following neoadjuvant AI was observed: 3011 of 13 278 total genes and 25 of 64 Stat3 target genes were differentially regulated following AI treatment (*p*-value = 0.004) (Fig. 5B). Again, 80% of the differentially regulated Stat3 target genes were regulated in a direction consistent with previous reports of the pathway being activated [29, 30, 33, 34]. This suggests that enhanced Stat3 activity and potential napabucasin sensitivity could be a feature of early responses to both tamoxifen and aromatase inhibitors.

Finally, using the same two patient datasets, we looked for evidence of altered Src activity in response to endocrine treatment. As Src is not a transcription factor, a list of target genes was not easily composed but a relevant gene list has previously been compiled, referred to as a dasatinib index [26]. Derived from in vitro cell responses to dasatinib, this gene panel has been used to try to predict sensitivity to dasatinib. There was no enrichment of the dasatinib index in the first patient dataset, following 21 days of tamoxifen treatment (*p*-value = 0.465) (Fig. 5A). A significant enrichment was however detected in the number of dasatinib index genes differentially regulated following 90 days of AI therapy (*p*-value = 0.002) (Fig. 5B). 88% of differentially regulated dasatinib index genes were regulated in a direction consistent with tumour tissue becoming more sensitive to dasatinib following neoadjuvant AI therapy [26]. This patient data analysis supports our in vitro findings that Stat3 and Src are differentially activated in the early weeks of tamoxifen treatment. Further investigation into the use of Stat3 inhibitors in particular, in combination with endocrine therapy in the neoadjuvant or adjuvant setting is warranted.

## Discussion

In summary, we present comprehensive proteomic profiling of MCF7 cells during long-term tamoxifen treatment. This resource is of high relevance to understanding the molecular basis of cancer progression and has direct bearing on the selection of companion drugs for future testing. Evident from changes in both growth patterns and protein expression, this population of cells underwent two distinct phases to adapt to tamoxifen treatment. We highlight the enhanced activity of Stat3 and enhanced sensitivity to Stat3 inhibitor napabucasin when tamoxifen was inhibiting growth and cells were in a dormant-like, pre-resistant phase. We demonstrate for the first time that this enhanced sensitivity happened shortly after initiation of endocrine therapy and not only when resistance had developed. This identifies potential for Stat3 inhibitor use in the adjuvant and neoadjuvant settings and not only in the advanced resistant setting.

Whether the acquisition of drug resistance occurs through subclonal expansion or cellular evolution remains an ongoing debate. It is our opinion that both processes likely play important roles in how cells adapt to develop tamoxifen resistance in our model system. Cellular plasticity is a common feature of breast cancer tissue [35]. A study of 27 strains of MCF7 cells has previously highlighted the extent of heterogeneity and clonal diversity as well as genomic instability that exist within MCF7 and other cell lines [36]. MCF7 cells are the preferred cell line model for studying endocrine resistance [37]. With advances in technology, elegant bar coding experiments have been conducted to monitor subclonal dynamics in many model systems as resistance develops. For example, Bhang and colleagues used barcoding to monitor erlotinib-resistant non-small cell lung cancer (NSCLC) cells and ALB1 inhibitor-resistant chronic myeloid leukemia (CML) cells. Their results indicate that the vast majority of resistant clones were selected from pre-existing subclones and were not de novo acquired [38]. Of note, drug resistance developed within a considerably shorter time frame (3–4 weeks for CML cells) for their models compared to ours. On the other hand, a reversible, drug-tolerant phenotype has been shown to emerge de novo from lung adenocarcinoma cells plated at low density [39]. Although it was outside the scope of our study, we expect that the initiation of high-dose tamoxifen would have enforced a selection pressure resulting in subclonal selection. The observation that growth rates took almost a year to stabilise and for resistance to be established suggests to us that these subclones were not inherently resistant to tamoxifen but at least some cells had the capacity to evolve into resistant subclones and be favoured for further selection. We believe it is unlikely that a coordinated, population-wide adaptation arose. Future studies to test the contribution of subclone dynamics versus population dynamics to the acquisition of resistance will be very important.

Stat3 has been gaining momentum as an important player in breast cancer progression. Induced by the cytokine IL-6, functional roles for Stat3 in inducing breast cancer growth [40] and driving breast cancer metastasis have recently been reported [41]. Enhanced Stat3 activity has been reported in established endocrine-resistant breast cancer cell models compared to endocrine-sensitive cells [42], leading to the testing of several Stat3 inhibitors in cell line and PDX models of endocrine-resistant and sensitive breast cancer [43, 44]. Indeed, Liu and colleagues detected enhanced sensitivity to napabucasin in an established cell line model of acquired tamoxifen resistance [44]. Our results concur with those previously reported but importantly, our data indicate that enhanced sensitivity to Stat3 inhibitors occurs prior to the establishment of endocrine resistance. Napabucasin is recognised as a stemness inhibitor which can block stem cell activity in cancer cells [45]. Conceptually, targeting stem cell activity prior to acquired resistance development is likely to have more impact on disease progression than targeting stem cell activity after resistance mechanisms have been established.

Napabucasin has been well tolerated in clinical trials and carries a favourable pharmacokinetic profile. The BRIGHTER phase III trial tested napabucasin in advanced gastric cancer in combination with paclitaxel. The trial was unblinded early due to the unlikelihood of reaching the primary overall survival endpoint. However, there were no safety concerns of clinical significance and the combination of napabucasin and paclitaxel was well tolerated [46]. The CanStem111P trial in advanced pancreatic cancer and the CanStem43L trial in advanced lung cancer were also stopped early due to futility or changes in standard of care but again no safety concerns were detected in these trials and both paclitaxel and gemcitabine were tolerated in combination with napabucasin. The CanStem303C trial in metastatic colorectal cancer reached completion but showed no survival benefit in the total population. A subset of patients with pStat3 positive tumours did respond significantly better to napabucasin than to placebo [47]. Adverse events were consistent with previous trials and FOLFIRI and bevacizumab were combined with napabucasin [48]. Thus, while the trials to date have not produced the desired outcomes, napabucasin shows good tolerability with capacity to be combined with multiple other drugs. Napabucasin and other Stat3 inhibitors hold great potential if optimal patient selection and timing can be identified.

Targeting Src to treat breast cancer is not a new concept. Src gene expression signatures have previously been linked with breast cancer bone metastases, independent of hormone receptor status [49]. Dasatinib is a tempting drug to examine as it is already licensed for use in chronic myeloid leukemia. Dasatinib has also previously been trialled and tolerated in multiple breast cancer trials, including in combination with endocrine therapy, in the advanced setting [25, 50]. Overall, results of these trials have been disappointing with response rates to dasatinib considerably lower than anticipated. It is a frustrating result, common to many Src inhibitors tested in many solid tumour types. There is strong evidence to indicate a central role for Src in the progression of many solid tumours and yet no Src inhibitor has been licensed for use in solid tumour malignancies. Nevertheless, it remains a focus area and the development of new inhibitors which are more specific for Src [51] or selection of the most appropriate patients may change that outcome over the next decade. Our results add important knowledge, indicating that Src targeting prior to endocrine resistance might be advantageous.

We have used Stat3 and Src as proof of principle that intelligent targeting of breast cancer cells prior to the development of endocrine resistance can be advantageous and enhance drug sensitivity. This study also highlights a number of other protein targets, which warrant further study, as listed in Tables 1 and 2. Of note, dynamic expression changes in Bcl2 family proteins would suggest that drugs such as venetoclax and navitoclax may be beneficial during the growth-impeded phase and warrant further exploration in this setting.

The concept that cells reach a tipping point in the development of resistance is gaining momentum [17, 18, 52]. Although we did not set out to identify such a tipping point, the data gathered clearly points to a biphasic shift in response to tamoxifen, which is consistent with the concept of a tipping point occurring at ~160 days in the MCF7 population studied. To the best of our knowledge, this is the first report of such a tipping point arising from a proteomic longitudinal study. The ability to retrospectively detect a tipping point, when cells transition to drug tolerance, by both proteomic and transcriptomic methods, encourages optimism that we may soon be able to detect this transition in real-time. As we expand our knowledge in the field, targeting cells in the growth-impeded phase is an exciting prospect with the potential to prevent the development of endocrine resistance. This study demonstrates that neoadjuvant endocrine treatment for 3 weeks or 3 months induces features of the growth-impeded phase. Window of opportunity trials in this neoadjuvant setting, where the response to treatment can rapidly be assessed, are therefore likely to be the best approach to introduce companion drugs to accompany endocrine therapy and prevent the development of endocrine resistance.

## Supplementary information

Supplementary Data

Supplementary Table S1

Supplementary Table S2

Supplementary Table S3

## Data Availability

All data generated or analysed during this study are included in this published article (and its supplementary information files).
