# Supplementary material for: Proteomic time course of breast cancer cells highlights enhanced sensitivity to Stat3 and Src inhibitors prior to endocrine resistance development
Source: Cancer Gene Ther. 2022 Oct 20;30(2):324–34. doi: 10.1038/s41417-022-00548-0 (PMC9935392; doi:10.1038/s41417-022-00548-0)
Supplement: Supplementary file 1 — Supplementary Data [file 41417_2022_548_MOESM1_ESM.pdf]

Supplementary Methods

Reverse Phase Protein Arrays (RPPA)

Protein extraction lysates were normalised to 1.5ug/ul concentration and 3 parts of cell lysates were mixed with 1 part of an SDS sample buffer (40% glycerol, 8% SDS, 0.25M Tris-HCl, pH6.8, 10% 2-mercaptoethanol). Samples were boiled and manually diluted in four-fold serial dilutions with lysis buffer. A 2470 Arrayer (Aushon BioSystem, MA, USA), created a sample array on Oncyte Avid nitrocellulose-coated slides (Grace Bio-Labs, OR, USA). The slides were stored with desiccant (Drierite, OH, USA) at −20 °C prior to immunostaining.

Immunostaining was performed on an automated slide stainer (Dako Link 48 – Agilent Technologies, CA, USA) according to the manufacturer's instructions. Each slide was incubated with a single primary antibody (see Supplementary Table S1 for details) at room temperature for 30 min. Secondary antibody was goat anti-rabbit IgG (1:5000) (Vector Laboratories, CA, USA) or rabbit anti-mouse IgG (1:10) (Agilent Technologies, CA, USA). Dako Secondary antibodies were used as a starting point for amplification via horseradish peroxidase-mediated biotinyl tyramide with chromogenic detection (diaminobenzidine) according to the manufacturer's instructions (Agilent Technologies, CA, USA).

Scanned TIFF images of slides were analyzed using Microvigene software version 5.1 (VigeneTech Inc., MA, USA) to generate spot signal intensities (Ruan, 2006). Instead of generating multiple linear regression curves for data quantification over each series of serial dilutions, the QRPPA module of Microvigene using a 4 parameter logistic-log model ("SuperCurve" algorithm (Hu et al., 2007)) that uses all spots within one array to form a sigmoid antigen-binding kinetic curve.

Finally the spots were normalized by protein loading using the entire panel of antibodies. Briefly, normalization is processed as follows: we determined the median for each antibody across the sample set and we divided each raw linear value by the median within each antibody to get the median-centered ratio. After that, we calculated the median from median-centered ratio for each sample across the entire panel of antibodies. This median functions as a correction factor (CF) for protein loading adjustment. We considered the samples an outlier if the CF is above 2.5 or below 0.25. Finally, we divided the raw data in linear value by the CF to obtain the normalized value.

Ruan M: Protein Microarray Image Analysis. In Functional Protein Microarrays in Drug Discovery. 1st edition. Edited by: Predki PF. CRC Press; 2006:359.

Hu J et al.: Non-parametric quantification of protein lysate arrays. Bioinformatics; 2007,15(23):1986-94.

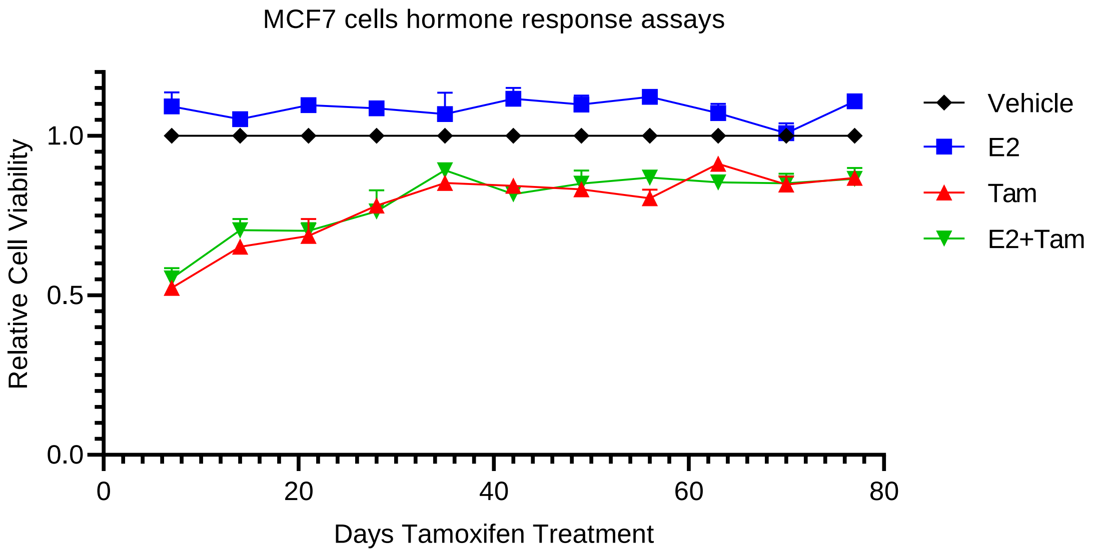

Supplementary Figure S1: Change in hormone responsiveness as cells adapt to long-term Tamoxifen treatment.

MCF7 cells were cultured long-term in the presence of 10^{-6}M 4-OHT. Each week a small number of cells were steroid depleted by culturing in 3% CDS media for 3 days, changing the media each day. Cells were seeded into 96 well plates with 5,000 cells per well. Hormone treatments were added as follows: Estradiol 10^{-8}M (E2), 4-hydroxy-Tamoxifen 10^{-6}M (Tam), the combination of 10^{-8}M E2 and 10^{-6}M 4-OHT (E2 +Tam) or Vehicle (0.1% ethanol). After 5 days MTS reagent was added and cell viability was calculated from absorbance readings at 490nm. Relative to vehicle, estrogen treatment consistently resulted in 10% more viable cells. Tamoxifen, or the combination of estrogen and Tamoxifen, initially resulted in almost 50% reduction in the number of viable cells. However, over the first 40 days of long-term culture in Tamoxifen, those cells surviving the culture conditions gradually lost sensitivity to Tamoxifen. From approx. 40 days onwards, Tamoxifen only induced a 10% reduction in cell viability. Each data point shows mean ± SEM with n=3.

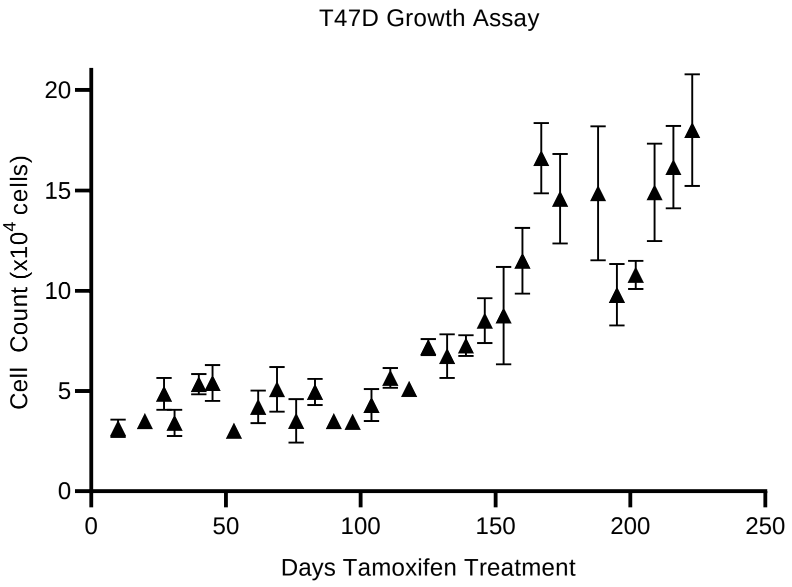

Supplementary Figure S2: Change in growth rate of T47D cells cultured long-term with Tamoxifen

T47D cells were cultured long-term in the presence of 10^{-6}M 4-OHT. Graph shows the results of 30 growth assays in T47D cells exposed to 10^{-6}M 4-OHT for varying times (0-250 days, x-axis) prior to initiation of the assay. On each occasion, 20,000 cells were seeded in triplicate wells and manual cell counts were conducted 7 days later (y-axis). Data points represent mean ± SEM with n=3 for each assay. The curve is a very similar shape to that for MCF7 cells but the timeframes are shorter. The Growth-Impeded Phase was determined as days 0-100 with the Growth Recovery Phase from days 101-250.
